# Editorial: Novel targets and state of the art therapies in ARDS and sepsis

**DOI:** 10.3389/fmed.2024.1496821

**Published:** 2024-11-05

**Authors:** Daniel O'Toole, Shahd Horie, Emma Murphy

**Affiliations:** ^1^Discipline of Physiology, University of Galway, Galway, Ireland; ^2^Discipline of Anaesthesia, University of Galway, Galway, Ireland; ^3^PRISM Research Institute, Technological University of the Shannon, Athlone, Ireland

**Keywords:** ARDS, sepsis, diagnostics, therapeutics, infection

Acute respiratory distress syndrome (ARDS) and sepsis remain leading causes of patient morbidity and mortality and the COVID-19 pandemic has highlighted the continuing lack of effective therapeutic options for these and other related acute inflammatory conditions. Among the problems facing ARDS researchers is that there is currently no specific biomarker for rapid diagnosis, and adhering to the Berlin consensus criteria (1) and therefore necessitates methods that are time consuming and expensive, particularly in the context of overloaded health care services. Recent hot topics are sub-phenotyping of patients, with clearly delineated hyper- and hypo-inflammatory versions of ARDS being more widely recognized (2) and emerging biomarkers of patient outcome giving clinicians the opportunity to treat these quite distinct disease variants with distinct therapeutic approaches. There are also still no licensed medicine specifically targeting ARDS or sepsis (3), a critical gap in the clinician's arsenal and individual organ and symptom support remains the mainstay (4).

Recently, a host of novel medicinal approaches have been investigated to address these problems, such as advances in the development of pharmacological agents, recombinant protein drugs, and cell and gene therapies. Bioinformatics based approaches and clinical profiling of patients are also paving the way for stratification, targeted therapies, and precision medicines. Here, we summarize breaking contributions to the field in a collection of articles published as part of the Research Topic entitled “*Novel targets and state of the art therapies in ARDS and sepsis*.”

Our first review paper explores the utility of measuring mitochondrial markers of ARDS related disease (McClintock et al.). The summarized studies include assessments of mitochondrial DNA in blood, peroxidation markers and a range of metabolites such as glucose, lactate and xanthine and point to a future where simple point of care devices could instantly diagnose ARDS and ARDS severity based on minimal essential parameters. In a patient sample analysis study, Peng et al. have identified dysfunctional iron metabolism mediated via hepcidin as a predictor of patient outcome in COVID-19 ARDS, a finding which could ultimately be applicable to ARDS of any etiology. Finally in this group of manuscripts we have a study of immune cell subpopulations in ARDS patients where it was discovered that the ratio of CD4/CD8 markers was an effective predictor of disease severity and could assist in directing resources and appropriate care to specific sufferers (Pascual-Dapena et al.).

Our second thematic grouping of papers is a deep dive into the pathology and pathobiology of ARDS. Indeed, it could be argued that this Research Topic overlaps with and informs diagnostics and therapeutics and is fundamental to an intelligent approach to ARDS patient care. As well as the alveolar cells of the lung, acute lung injury is also associated with endothelial dysfunction and vascular thrombosis and we are provided with a comprehensive overview of how the Kallikrein-Kinin axis contributes to this disease process (Bailey et al.). Large datasets demand increasingly complex computational approaches to maximize the meaningful information extracted, and so we are happy to welcome from Parkinson et al. a machine learning assisted mRNA profiling of one of the more vulnerable patient populations, that of neonatal sepsis. We also see a single-cell analysis approach to assessment of risk factors for progression of shock to ARDS that has identified the importance of chromatin accessibility near a specific gene locus, CALCRL (Armstead et al.). To round off this section, we have two studies focusing on specific disease mechanisms and their involvement in ARDS and sepsis. Firstly, Liu et al. elucidates the contribution of the ferroptosis pathway in ischemia/reperfusion driven inflammation in a rat model and secondly we have from Hu et al. an intriguing paper detailing the involvement of the C-type lectin pancreatic stone protein in multiple organ dysfunction syndrome (MODS).

In our final subsection we explore approaches to ARDS and sepsis patient management and therapeutics, from refining traditional support protocols to cutting edge advanced therapeutic medicinal products (ATMPs). This theme includes a retrospective sepsis patient analysis comparing saline and Ringers solutions for resuscitation (Isha et al.), followed with a preclinical study from González et al. of a nebulizer delivered stem cell therapy for ARDS, this with the novelty of utilizing the secretome as opposed to the cell itself.

We, the editors of this special edition of Frontiers in Medicine, hope that you, the reader, find this Research Topic to be as informative and interesting as we did when assembling and curating it, and expect that it will spark future research into diagnosing and treating this devastating family of diseases.
